# Tracking the Risk of Cardiovascular Disease after Almond and Oat Milk Intervene or Statin Medication with a Powerful Reflex SH-SAW POCT Platform

**DOI:** 10.3390/s24206517

**Published:** 2024-10-10

**Authors:** Chia-Hsuan Cheng, Hiromi Yatsuda, Han-Hsiang Chen, Guang-Huar Young, Szu-Heng Liu, Robert YL Wang

**Affiliations:** 1 Graduate School of Science and Technology, Shizuoka University, 3-5-1 Johoku, Naka-ku, Hamama-tsu-shi 432-8561, Japan; joshcheng@tst.bio (C.-H.C.); yatsuda.hiromi@tst.bio (H.Y.); 2 tst Biomedical Electronics Co., Ltd., Taoyuan 324403, Taiwan; 3 Department of Biomedical Sciences, College of Medicine, Chang Gung University, Taoyuan 33302, Taiwan; h318joe@gmail.com (H.-H.C.); youngguanghuar@gmail.com (G.-H.Y.); 4 Kidney Research Center and Department of Nephrology, Chang Gung Memorial Hospital, Linkou 33305, Taiwan; 5 Division of Pediatric Infectious Diseases, Department of Pediatrics, Chang Gung Memorial and Children’s Hospital, Linkou 33305, Taiwan

**Keywords:** SH-SAW, biosensor, POCT, cardiovascular disease (CVD), almond, oat milk, statin

## Abstract

Cardiovascular disease (CVD) represents the leading cause of death worldwide. For individuals at elevated risk for cardiovascular disease, early detection and monitoring of lipid status is imperative. The majority of lipid measurements conducted in hospital settings employ optical detection, which necessitates the use of relatively large-sized detection machines. It is, therefore, necessary to develop point-of-care testing (POCT) for lipoprotein in order to monitor CVD. To enhance the management and surveillance of CVD, this study sought to develop a POCT approach for apolipoprotein B (ApoB) utilizing a shear horizontal surface acoustic wave (SH-SAW) platform to assess the risk of heart disease. The platform employs a reflective SH-SAW sensor to reduce the sensor size and enhance the phase-shifted signals. In this study, the platform was utilized to monitor the impact of a weekly almond and oat milk or statins intervention on alterations in CVD risk. The SH-SAW ApoB test exhibited a linear range of 0 to 212 mg/dL, and a coefficient correlation (R) of 0.9912. Following a four-week intervention period, both the almond and oat milk intervention (−23.3%, *p* < 0.05) and statin treatment (−53.1%, *p* < 0.01) were observed to significantly reduce ApoB levels. These findings suggest that the SH-SAW POCT device may prove a valuable tool for monitoring CVD risk, particularly during routine daily or weekly follow-up visits.

## 1. Introduction

Cardiovascular disease (CVD) is a collective term used to describe a group of pathological conditions that affect the heart and blood vessels. These include, but are not limited to, coronary artery disease, heart failure, and stroke. CVD is the leading cause of mortality worldwide for both men and women and across most racial and ethnic groups. In 2020, it was estimated that 19 million individuals died from CVD globally, representing an 18.7% increase from 2010 [1,2]. The early detection and intervention of CVD are essential for the control of the disease and the prevention of serious complications. The monitoring of CVD entails the observation of risk factors, including elevated high blood pressure, cholesterol, and blood sugar levels, as well as the assessment of lifestyle habits such as tobacco use, physical activity, and dietary patterns. Regular physical examinations, blood tests, and imaging tests facilitate the early detection of CVD, thereby enabling prompt treatment and a more efficacious intervention [3]. A point-of-care electrocardiogram (POCT) is an assessment of a patient’s cardiac health and function conducted at the time of treatment, as opposed to sending samples or patients to a centralized laboratory or diagnostic facility. This approach facilitates expedited diagnosis and treatment decisions, particularly in emergency settings. Point-of-care testing (POCT) represents an efficacious methodology for monitoring the progression of cardiovascular status. In contrast to electrocardiography (ECG) or point-of-care ultrasound (POCUS), POCT is more cost-effective on an individual basis and does not necessitate the expertise of a trained professional to interpret the results. Furthermore, POCT has the potential to positively impact health outcomes, particularly in remote, rural, and underserved communities [4]. The Cholestech LDX Analyzer (Abbott, Chicago, IL, USA), the cobas^®^ b 101 system (Roche, Basel, Switzerland), and PixoTest (iXensor, Taipei, Taiwan) are widely utilized point-of-care testing devices that can rapidly test blood lipids and measure total cholesterol, LDL, HDL, and triglycerides. However, there is currently a dearth of point-of-care devices on the market that are specifically designed to measure apolipoprotein B.

Statins represent a class of pharmaceutical drugs that are frequently utilized for the treatment of high blood cholesterol levels, a condition that is a significant risk factor for CVD. Statins reduce the amount of cholesterol in the blood by inhibiting 3-hydroxy-3-methylglutaryl coenzyme A (HMG-CoA) reductase in the liver, an enzyme that plays a pivotal role in cholesterol production [5]. By reducing elevated levels of low-density lipoprotein cholesterol (LDL-C), or “bad” cholesterol, statins assist in the prevention of atherosclerosis, a process characterized by the accumulation of plaque within the arteries. Statins represent an efficacious intervention for the management of elevated cholesterol and the reduction of CVD risk [6,7]. Statins elicit a reduction in LDL-C levels within a few weeks of initiating treatment, with studies demonstrating that LDL-C levels can typically be diminished by 25% to 60% [7].

Another potential avenue for the management of LDL cholesterol levels in the blood is food therapy. A number of studies have demonstrated that almonds may help to reduce the risk of developing CVD. Almonds are a rich source of monounsaturated and polyunsaturated fats, as well as antioxidants. These nutrients have been shown to contribute to the reduction of LDL-C levels in the blood, while also exhibiting anti-inflammatory properties. Chronic inflammation is a significant contributing factor to the development of CVD. Reducing inflammation can, therefore, help to lower the risk of heart disease and other related conditions. Studies have demonstrated that consuming nuts can result in a reduction of LDL by 3–19% [8,9]. Oat milk is a rich source of soluble fiber oat beta-glucan, which has been shown to assist in lowering LDL-C and apolipoprotein B (ApoB) levels in the blood [10,11].

To assess the risk of CVD, regular monitoring of blood tests using POCT devices shows great promise. A shear horizontal surface acoustic wave (SH-SAW) biosensor system is one of the attractive candidates. SH-SAW biosensors offer several advantages, particularly in terms of detecting biomolecular interactions and sensing in the liquid environments, which are often used for point-of-care diagnostics. Compared to other types of sound waves (e.g., Rayleigh waves), SH-SAW is less affected by liquid attenuation. This makes it ideal for biosensing applications in liquids, such as blood, serum, or other biological fluids, where the energy of the acoustic waves can be retained at the sensor surface, resulting in a more accurate reading [12,13]. In this study, we employed a SH-SAW biosensor system comprising a palm-sized reader and a disposal cartridge containing a 250 MHz SH-SAW sensor chip [14]. The inter-digital transducers (IDTs) serve to convert the electrical input signal into a measurement circuit. To prevent short circuits resulting from contact with specimen liquids, the IDTs are encased in an epoxy wall structure and safeguarded by a glass cover. The sensing area measures 2000 μm in length, with a 4000 μm round trip, corresponding to 100 and 200 wavelengths, respectively. Positioned between the IDTs and reflectors, the sensing area is coated with capture antibodies designed to detect the target substances [15,16]. The SH-SAW design employs unidirectional IDTs and reflective delay lines, which, in conjunction with the reflectors, can enhance sensitivity and miniaturize the point-of-care diagnostic platform. Their compact size and multiplexing potential make them ideal for portable diagnostic devices. The system is designed to detect alterations in the physical characteristics of immune binding on the surface of the sensor. By measuring the change in the propagation of an acoustic wave signal on the sensor surface, the SH-SAW system is capable of accurately measuring the level of apolipoprotein B in real time [14].

ApoB is a protein that is found in lipoproteins, which are particles in the blood that are responsible for the transportation of cholesterol and other lipids. ApoB is the primary protein constituent of LDL particles, and elevated levels of ApoB are significantly correlated with an increased risk of CVD. The measurement of ApoB levels provides a more accurate assessment of CVD risk than the measurement of LDL-C levels alone. This is because LDL levels do not reflect the number of LDL particles in the blood [17]. This is important because individuals with identical LDL cholesterol levels may exhibit disparate numbers of LDL particles. Those with elevated numbers of LDL particles are at an elevated risk of CVD. In clinical practice, cholesterol-lowering therapies (such as statins) may target ApoB levels and function by reducing the production of ApoB-containing particles in the liver. A reduction in apolipoprotein B (ApoB) levels has been demonstrated to diminish the likelihood of developing CVD. Furthermore, the monitoring of ApoB levels enables clinicians to evaluate the efficacy of cholesterol-lowering therapies and make necessary adjustments to treatment plans. The ApoB SH-SAW biosensor plays a pivotal role in the early detection and monitoring of CVD, facilitating prompt intervention and treatment.

In this paper, we developed an ApoB POCT device based on the SH-SAW platform to evaluate the effects of statin, almonds, and oat milk on reducing ApoB concentrations. We also introduce a POCT platform that tracks cardiovascular disease risk on a weekly basis, providing a new technique for in-depth assessment of cardiovascular disease risk in the clinical setting.

## 2. Materials and Methods

### 2.1. Materials

iProtin immunoassay reader (BMREDE0101) and SH-SAW biosensor bare chips (TN0100R) were supplied by tst biomedical electronics Co., Ltd. (Taoyuan, Taiwan). Phosphate-buffered saline (CWFF0613) and bovine albumin serum (FUBSA001.100) were bought from Bio-Future company (BioFuture biotech, Taoyuan, Taiwan). Dithiobis [succinimidyl propionate] (DSP) was purchased from Thermo Fisher Scientific (Waltham, MA, USA) Dimethyl sulfoxide (DMSO) was purchased from Sigma-Aldrich. The stabilizer was prepared by adding 15 µL of Tween 20 to 10% sucrose solution (BCBV9208, Sigma, St. Louis, MO, USA). The ApoB monoclonal antibody (MBS531324) was purchased from MyBioSource (San Diego, CA, USA) and the ApoB calibrator (ODR3022) was purchased from Beckman coulter (Brea, CA, USA).

### 2.2. Fabrication of ApoB SH-SAW Biosensor

**Figure 1:**
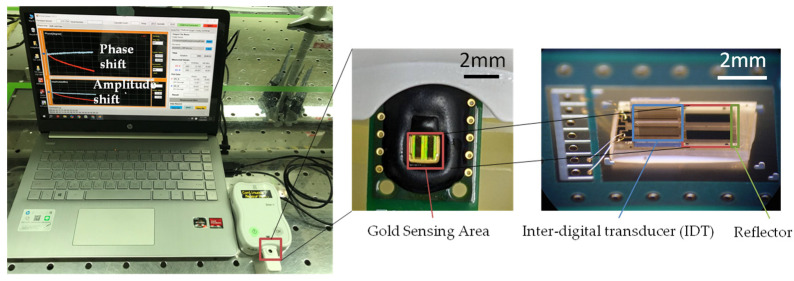
The reflective SH-SAW biosensor chip employs a measurement system and structure comprising a forked finger sensor (IDT), a gold sensing area, and a reflector.

The SH-SAW POCT platform provides a 3 mm × 5 mm dual-channel SH-SAW biosensor chip, as shown in Figure 1; one channel for reference and the other for capture. The substrate material for the chip is 36Y-cut 90X-propagation quartz with a 0.35 mm thickness. Each channel on the chip has an IDT with a center frequency of 250 MHz, a sensing region with an approximately 100 nm thick gold film, and a reflector. To prevent short-circuiting from contact with specimen liquids, the IDTs are enclosed by an epoxy wall structure and protected by a glass lid. The sensing area measures 2000 μm in length with a 4000 μm round trip, corresponding to 100 and 200 wavelengths, respectively. The process of biomaterial coating was as follows: Firstly, the gold sensing area of the chip was cleaned with 2% of Hellmanex III. Then, the chip was washed twice with distilled water. We then added 0.4 mg/mL of DSP solution in DMSO and incubated for 5 min. DSP cross-linker self-assembled with the gold sensing area on one side and the amine group of the biomaterial on the another. After that, the chip was rinsed with DMSO and washed two times with distilled water to remove excess cross-linker. The sensing area was coated with 0.12 mg/mL of ApoB monoclonal antibody. After that, the chips were blocked with 2% bovine serum albumin to fill the un-coated area. Finally, a stabilizer was added for long-term storage. The coated chips were used to establish four-parameter logistics (4PL) standard curves [14].

### 2.3. Performance of ApoB SH-SAW Biosensor

The performance of the ApoB SH-SAW biosensor was evaluated with Clinical and Laboratory Standards Institute (CLSI) Guidelines [18]. The limit of blank (LoB) and limit of detection (LoD) are parameters to confirm the sensitivity of the biosensor by performing sixty replicate tests at low concentrations to check the standard deviation and coefficient of variation. The precision of the ApoB biosensor was evaluated by conducting 20 repeated measurements with whole blood samples on the same day and continuous measurements over 5 days. The comparison study was conducted with commercialized ApoB product. In total, 154 whole blood samples were used to calculate the slope and coefficient of correlation (R).

### 2.4. Measurement of ApoB by SH-SAW Biosensor

**Figure 2:**
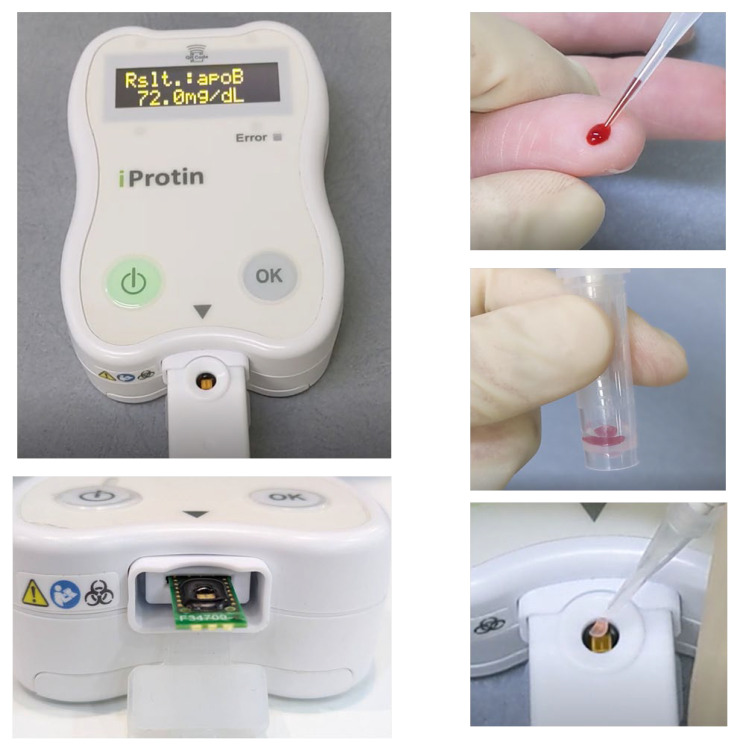
Operation of the iProtin reader with ApoB SH-SAW biosensor.

Blood samples were collected from the subject’s fingertips. A 5 μL blood sample was collected with a micropipette and mixed with dilution buffer in a test tube. The diluted sample was then dropped onto the ApoB SH-SAW biosensor (Figure 2). ApoB was captured by the pre-immobilized anti-ApoB monoclonal antibody on the biosensor surface. When ApoB particles in the sample bind to the antibodies on the SH-SAW biosensor, the velocity of the acoustic waves changes. This alteration in the acoustic properties of the biosensor is then detected and measured by the reader. iProtin Reader captures this change to determine the concentration of ApoB in whole blood with a 4PL standard curve [14].

### 2.5. Study Design and Participant

**Figure 3:**
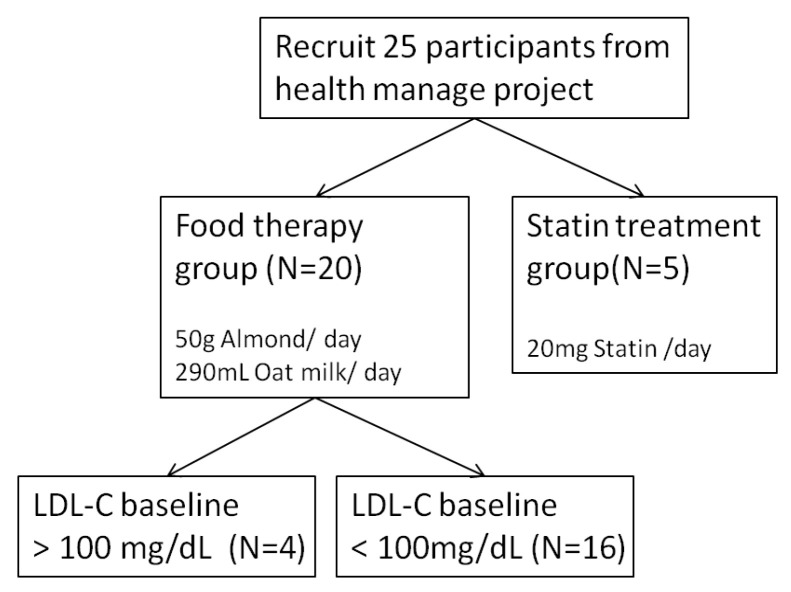
Recruitment and categorization of participants.

**Figure 4:**
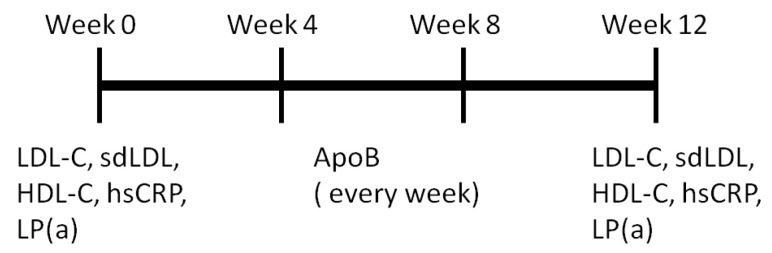
Schedule for measuring cardiovascular indices.

Participants were recruited and screened for eligibility through the Health Management Program (HMP), a collaboration between Tai-Saw Technology CO., LTD (Taoyuan, Taiwan) and the Department of Family Medicine at Chang Gung Memorial Hospital, in accordance with the guidelines approved by the institutional review board of Chang Gung Memorial Hospital in Taiwan (IRB No. 201601701B0). Written informed consent was obtained from all subjects. Subjects who met the inclusion criteria were assigned to either the food therapy (50 g/day almonds and 290 mL/day oat milk) group or the statin treatment (20 mg/day) group (Figure 3). All the subjects in the statin treatment group were diagnosed by a physician as needing medication. Twenty-five of these subjects were followed up weekly during the 12 weeks of food therapy or statin treatment to assess ApoB concentrations using the SH-SAW POCT. Low-density lipoprotein cholesterol (LDL-C), high-density lipoprotein cholesterol (HDL-C), high-sensitivity C-reactive protein (hsCRP), and lipoprotein(a) (Lp(a)) were measured before and after 12 weeks of intervention using the Tba 200fr Neo automated clinical chemistry analyzer provided by Toshiba medical (Tokyo, Japan) (Figure 4).

### 2.6. Statistical Methods

All data in this study are expressed as means ± SD (standard deviation). Paired-samples *t*-tests showed significant differences before and after the food therapy or statin treatment interventions. The *p*-value was less than 0.05, suggesting that the intervention was associated with a greater reduction in concentration of ApoB.

## 3. Results

**Figure 5:**
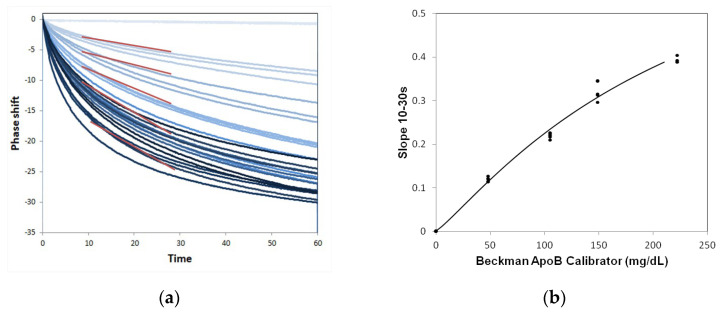
Establishment of 4PL fitting curve. (**a**) The real time curves of various ApoB concentrations. The darker line represents a higher concentration, the red lines indicate the slope at 10–30 s, which were used to establish the 4PL fitting curve; (**b**) 4PL fitting curve of ApoB chips based on the 10–30 s slope of the real-time curve.

Figure 5a illustrates the slope (red line) of the real-time phase change curves for varying concentrations of the ApoB standards. The slope facilitates the quantitative detection of ApoB from 0 to 212 mg/dL at 10 to 30 s. The standard curve is calculated from these slopes using four-parameter logistics (4PL). To create the standard curve, these slope signals from different concentrations of the calibrant are fitted to the following 4PL equation:

(1) $$[y]=D+\frac{A-D}{1+([x]\;/\;C^{B})}$$

where A = 0.0014, B = 1.4748, C = 117.33, and D = 0.5784 are the coefficients for the ApoB biosensor chip with R of 0.9947; [x] is the concentration of the calibrator; and [y] is the slope signal from the real-time curve, as shown in Figure 5.

**Figure 6:**
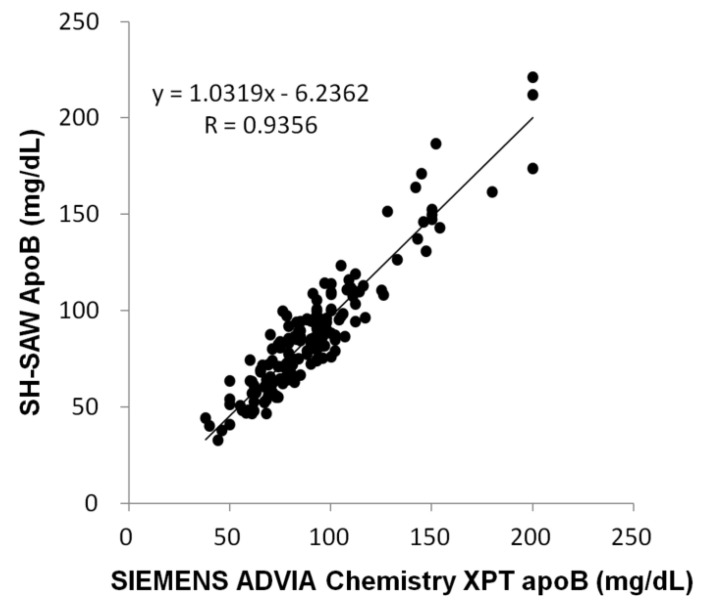
The comparison study of the SH-SAW biosensor and the commercially available product.

The limit of blank (LoB) (5.47 mg/dL) and limit of detection (LoD) (8.01 mg/dL) were determined by calculating the standard deviation and coefficient of variation from 60 replicate tests at low concentrations [18]. Within-run repeatability and between-run repeatability precision coefficients of variation (CV) were less than 10% at ApoB concentrations between 42 and 120 mg/dL. A comparison study was conducted using a commercially available ApoB detection kit with all 154 whole blood samples. The slope of the comparison of measurement (1.0319) and the correlation coefficient (R, 0.9356) suggest a high degree of agreement between the two test methods (Figure 6).

A total of 25 participants provided data for the health management project. As illustrated in Table 1, the two subject groups received disparate interventions to regulate blood lipid levels and cardiovascular risk factors. One group employed a dietary approach, particularly incorporating almonds and oat milk, while the other group utilized statin therapy. The decision-making process for assigning patients to the food therapy or statin treatment group was based on a number of factors, including LDL-C levels (>130 mg/dL) and the patients’ willingness to take medication. Twenty participants were assigned to the therapeutic food therapy group, with a baseline ApoB value of 91.56 ± 26.49 mg/dL. The baseline HDL-C value was 59.60 ± 19.58 mg/dL. The baseline of LDL-C value was 135.9 ± 39.38 mg/dL. The baseline sdLDL value was 38.85 ± 23.42 mg/dL. The baseline hsCRP value was 0.34 ± 0.40 mg/dL. The baseline Lp(a) value was 33.08 ± 42.47 mg/dL. The remaining five participants were assigned to the statin group, with baseline ApoB values of 85.83 ± 13.82 mg/dL, baseline HDL-C values of 46.80 ± 6.87 mg/dL, and baseline LDL-C values of 167.8 ± 45.60 mg/dL. The baseline concentration of sdLDL was 63.76 ± 21.85 mg/dL, while the baseline concentration of hsCRP was 0.16 ± 0.20 mg/dL. The baseline concentration of Lp(a) was 20.47 ± 23.05 mg/dL.

**Figure 7:**
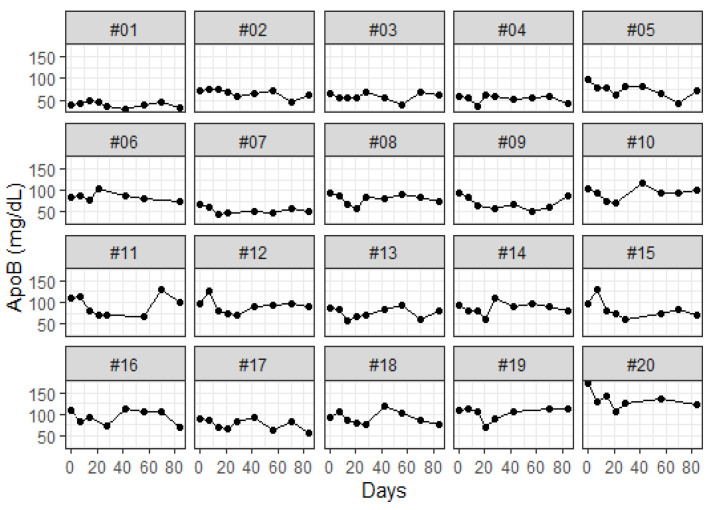
Follow-up of ApoB concentration after 12 weeks of food therapy intervention.

As illustrated in Figure 7, the SH-SAW biosensor was utilized to quantify the ApoB concentrations of each subject. Table 2 provides a summary of the overall cardiovascular index. Following a 12-week food therapy intervention, there was a statistically significant reduction in ApoB levels by 16.97% (76.02 ± 22.52 mg/dL, *p* < 0.001). Additionally, LDL-C levels demonstrated a 8.60% (124.2 ± 35.22 mg/dL, *p* < 0.01) reduction, and sdLDL also demonstrated a statistically significant decrease of 28.75% (27.68 ± 16.58 mg/dL, *p* < 0.001) after 12 weeks of food therapy. However, no significant differences were observed between the baseline and 12-week dietary therapy periods for HDL-C (59.2 ± 18.7 mg/dL, *p* = 0.338), hsCRP (0.2 ± 0.23 mg/dL, *p* = 0.063), and Lp(a) (26.63 ± 25.37 mg/dL, *p* = 0.089). The sustained decreases in ApoB and LDL-C indicate that almond and oat milk contribute to the reduction of low-density lipoproteins or larger lipoproteins. However, the decreases in HDL and LDL cholesterol are smaller.

**Figure 8:**
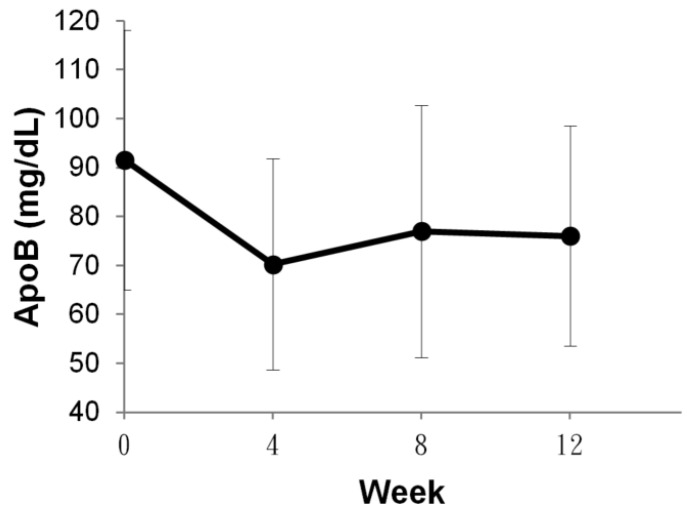
The change of ApoB concentrations at 4, 8, and 12 weeks after the food therapy.

As shown in Figure 8, the data demonstrate a statistically significant reduction in ApoB after 4 weeks (70.22 ± 21.56, *p* < 0.001), 8 weeks (76.95 ± 25.77, *p* < 0.05), and 12 weeks (76.02 ± 22.52, *p* < 0.001) of food therapy. The ApoB concentration exhibited a reduction of 23.3%, 15.96%, and 16.97%, respectively, in comparison to the pre-intervention period (Figure 8).

**Figure 9:**
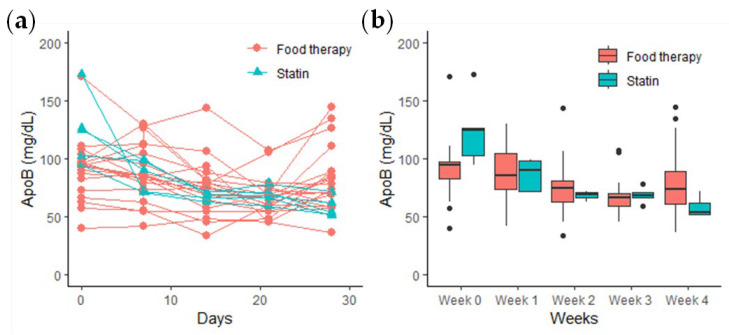
Comparison of ApoB-lowering effects in the food therapy group versus the statin treatment group: (**a**) line chart; (**b**) box plot.

A comparative analysis of the efficacy of the food therapy group and statin treatment group in mitigating the risk of cardiovascular disease revealed that the food therapy group exhibited a reduction in ApoB levels at 2 weeks (74.96 ± 23.59 mg/dL, *p* < 0.05) and 4 weeks (70.22 ± 21.56 mg/dL, *p* < 0.05) after the start of the intervention (Figure 9). In contrast, the statin treatment group demonstrated a notable reduction in ApoB levels after the first week (85.83 ± 13.82 mg/dL, *p* < 0.05) and fourth week (58.12 ± 8.49 mg/dL, *p* < 0.01) following the intervention. The upper and lower quartiles exhibited a tendency to converge in the second and third weeks following the intervention’s commencement, indicating that the ApoB levels within the groups gradually reached a similar level and that the variability within each group decreased.

**Figure 10:**
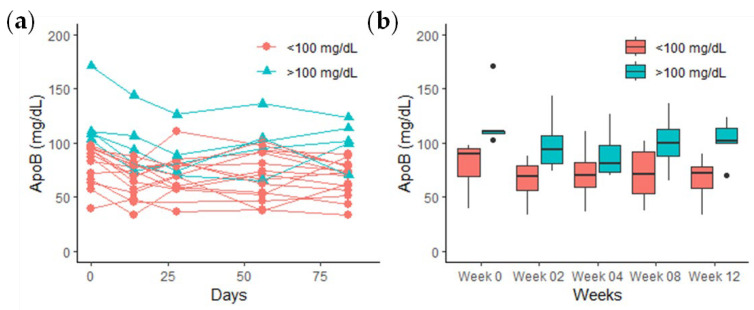
Comparison of the effect of food therapy group in reducing the high-ApoB-baseline (>100 mg/dL) and the low-ApoB-baseline (<100 mg/dL) groups: (**a**) line chart; (**b**) box plot.

To ascertain the impact of food therapy on the reduction of cardiovascular disease risk at varying baseline levels of ApoB, the food therapy group was subdivided into an ApoB high-baseline group (>100 mg/dL) and an ApoB low-baseline group (<100 mg/dL). In comparison to the baseline levels at week 0 (81.82 ± 17.85 mg/dL), the low-ApoB-baseline group exhibited significantly reduced ApoB levels at week 4 (71.72 ± 20.07 mg/dL, *p* < 0.05) and week 12 (67.43 ± 16.07 mg/dL, *p* < 0.01) (Figure 10). The high-ApoB-baseline group exhibited a significant reduction at week 4 (85.79 ± 23.90 mg/dL, *p* < 0.001) and week 0 (120.75 ± 28.22 mg/dL). Nevertheless, no significant difference was observed between week 12 (101.79 ± 19.95 mg/dL, *p* = 0.0656) and week 0 (120.75 ± 28.22 mg/dL).

## 4. Discussion

The World Health Organization (WHO) has established a set of criteria, known as the “ASSURED” criteria, that newly developed POCT devices must meet in order to be considered suitable for use. These criteria are as follows: affordable, sensitive, specific, user-friendly, rapid, robust, device-independent, and ready for delivery to the end user [19]. A POCT device is used in proximity to the patient, facilitating the delivery of results in a relatively brief timeframe, ideally within a range of 10 to 20 min [20]. The SH-SAW platform enables the completion of rapid tests in less than a minute through the calculation of the slope of a real-time curve for a specific standard range. The signal can be analyzed using the end-point window, which is defined as the phase shift signal at the initial baseline point minus the phase shift at a specific time point. However, endpoint data are more susceptible to an incorrect initial point and are often saturated at high concentrations. An alternative approach to the analysis of real-time data is the utilization of the slope of a specific real-time curve range. The advantage of the slope window is that a rapid result can be obtained within a few seconds [14]. In this study, the ApoB SH-SAW POCT employs a slope range of 10–30 s, enabling the completion of the test within 30 s.

The reflective SH-SAW sensor comprises an inter-digital transducer (IDT) and a reflector, and can be miniaturized, in contrast to the two IDT type SH-SAW sensors. Furthermore, the propagation of acoustic waves in both directions along the delay line allows for the acquisition of twice the signal. The substrate utilized in this study, with dimensions of 3 × 5 mm, is notably smaller than that employed in other studies, which ranged from 1 × 1 cm to 10 × 10 cm [21]. Overall, this newly designed, smaller device is well-suited for home care or rural areas.

The applications of POCT are developing rapidly, and it can rapidly obtain results with minimal interference before analysis, thereby improving patient outcomes [20]. POCT for cardiovascular disease biomarkers is applicable in a variety of settings, including home medical facilities, emergency departments, at the patient’s bedside, and rural clinics [22].

A significant number of point-of-care tests (POCTs) in the field of cardiology concentrate on the evaluation of acute coronary syndrome, with cardiac troponin tests representing a prominent example [20,21]. It is crucial to monitor lipoprotein status at an early stage, particularly in patients with an elevated risk of cardiovascular disease [23,24]. The utilization of POCTs in cardiology prior to primary care visits is a promising approach that is both acceptable and feasible. Furthermore, it has the potential to facilitate the initiation of statin therapy, which is recommended by guidelines for high-risk adults [25].

SAW technology has been employed for the detection of a range of markers, including CRP [14,26], antibodies associated with SARS-CoV-2 [27,28], and lipoproteins [14,29]. For comprehensive monitoring of cardiovascular disease risk, the SH-SAW biosensor offers a convenient platform for individual tracking. SH-SAW biosensors are capable of detecting biomolecules without the use of markers or labels, thereby enabling the measurement of whole blood in a drop-by-drop manner [14,29]. Moreover, the SH-SAW biosensor can be manufactured on compact and cost-effective substrates, which makes it well-suited for incorporation into portable or handheld devices [14,27]. In addition, ApoB is a valuable biomarker for evaluating CVD risk, as it reflects the number of LDL particles in the blood, not merely the level of LDL cholesterol. Even when LDL cholesterol levels are within the normal range, individuals with elevated ApoB levels are at an increased risk of developing CVD [17,30,31]. An elevated number of ApoB particles within the arterial lumen correlates with an increased likelihood of ApoB particles being retained within the arterial wall, resulting in augmented arterial damage. Conversely, a reduction in the number of ApoB particles through treatment can mitigate arterial damage and enhance the potential for healing [32]. The ApoB SH-SAW biosensor has garnered significant interest for its capacity to detect and monitor CVD at an early stage, facilitating timely intervention and treatment.

The results of this study demonstrated that following 12 weeks of daily consumption of 50 g of almonds and 290 milliliters of oat milk as a dietary supplement, there was a 16.97% reduction in ApoB levels and an 8.60% reduction in LDL-C levels compared to the baseline. These findings are consistent with the study that demonstrated that high-fat patients who consumed 73 g of almonds per day could reduce their LDL cholesterol levels by 9.4% compared to baseline [9]. Kalita et al. suggest that consuming approximately 45 g of almonds per day can assist in reducing one of the most significant risk factors for cardiovascular disease. Incorporating whole almonds into the diet represents a safe and practical nutritional strategy for controlling dyslipidemia [33].

As with other almond studies [34], almond dietary therapy did not result in a statistically significant alteration in HDL-C, Lp(a), or hsCRP. The cholesterol-lowering effect of almonds may be attributed to their high content of unsaturated fatty acids, fiber, and protein, which facilitate the reduction of cholesterol and bile acid absorption and reabsorption, enhance bile acid and cholesterol excretion, and augment LDL-C receptor activity [8,9,35].

The reduction of LDL cholesterol levels by statins typically occurs within a few weeks of initiating treatment. A reduction in LDL cholesterol of between 25% and 61% has been demonstrated in patients taking 2.5 to 80 mg of atorvastatin daily [7]. In our study, the statin treatment group was able to significantly reduce ApoB levels from baseline by 30.74% and 53.11% in the first and fourth weeks, which is consistent with the results of previous study.

The most effective method for reducing blood levels of ApoB or LDL-C remains statin therapy. A study conducted over a four-week period demonstrated that statin therapy was twice as effective as dietary therapy in reducing ApoB levels. Additionally, research has shown that consuming 100 g of almonds per day can significantly reduce non-HDL-C levels in individuals undergoing statin therapy, when compared to those receiving statin therapy alone [35].

Additionally, the rate at which statins reduce ApoB levels may be contingent upon the initial ApoB levels. Patients with higher initial ApoB levels may experience a more rapid decline in ApoB levels following statin therapy. However, a rebound was observed in the group with higher baseline ApoB levels, and the increase in ApoB levels may be attributed to poor compliance in the late stages of the study, as well as the necessity for further studies in a limited number of subjects to confirm these findings. Some participants expressed concern that the caloric intake associated with the dietary therapy would result in weight gain. In a study by Liu et al., it was found that moderate intake of almonds before meals can reduce visceral fat levels, body fat mass, and body fat percentage when compared to an isocaloric high-carbohydrate cookie. However, no change was observed in body weight [36]. Randomized controlled trials have demonstrated that almonds lead to a modest yet statistically significant mean reduction in body weight and fat content compared to a control diet. This helps to reduce the risk of overweight or obesity, particularly when the dose exceeds 42.5 g/day and lasts for more than six weeks [37].

While SH-SAW biosensors offer high sensitivity and accuracy, they also have inherent limitations. A significant limitation is that the manufacturing process for SH-SAW devices can be complex and costly, which may impede their widespread adoption, particularly in resource-limited settings. In contrast, POCT lateral flow assays are generally more cost-effective and easier to use, without the need for specialized equipment or training. Both SH-SAW and lateral flow POCT technologies offer valuable advantages in their respective applications, though they differ in terms of sensitivity, complexity, cost, and time to results. SH-SAW technology is particularly well-suited to high-sensitivity and quantitative biosensing applications, while POCT lateral flow assays are ideal for rapid and convenient point-of-care testing.

## 5. Conclusions

The reflective SH-SAW-based ApoB biosensor offers a practical and efficient platform for the monitoring of CVD risk and the evaluation of the effectiveness of various treatments. The regular consumption of almond and oat milk was found to significantly reduce levels of ApoB, LDL-C, and sdLDL However, this did not result in any change in the levels of HDL-C, Lp(a), or hsCRP. It should be noted that statin therapy remains the most effective intervention for reducing levels of ApoB and LDL-C. This study underscores the potential of the SH-SAW POCT device for routine CVD risk tracking and personalized disease management. To enhance usability and further optimize its performance, future work would focus on integrating microfluidic designs into the sensor chip to streamline sample processing and facilitate the more widespread applicability of the SH-SAW platform in point-of-care settings.

## Figures and Tables

**Table 1:** Participants’ information and baseline values for cardiovascular indices.

	Group	
	Food Therapy	Statin Treatment
Subject	20	5
Gender	10 Male; 10 Female	5 Male
Intervene	Almond (50 g/day) & oat milk (290 mL/day)	Statin (20 mg/day)
Baseline		
ApoB	91.56 ± 26.49	85.83 ± 13.82
HDL-C	59.60 ± 19.58	46.80 ± 6.87
LDL-C	135.9 ± 39.38	167.8 ± 45.60
sdLDL	38.85 ± 23.42	63.76 ± 21.85
hsCRP	0.34 ± 0.40	0.16 ± 0.20
Lp(a)	33.08 ± 42.47	20.47 ± 23.05

**Table 2:** The risk of cardiovascular disease after 12 weeks of food therapy.

Marker	Week 0	Week 12	*p* Value
ApoB	91.56 ± 26.49	76.02 ± 22.52	*p* < 0.001
HDL-C	59.60 ± 19.58	59.20 ± 18.70	N.S
LDL-C	135.90 ± 39.38	124.20 ± 35.22	*p* < 0.01
sdLDL	38.85 ± 23.42	27.68 ± 16.58	*p* < 0.001
hsCRP	0.34 ± 0.40	0.20 ± 0.23	N.S
Lp(a)	33.08 ± 42.47	26.63 ± 25.37	N.S

## Data Availability

The datasets used and analyzed during the current study are available from the corresponding authors upon reasonable request.
